# Knowledge of bloodless medicine among nurses at the Medical/Surgical Directorate of Komfo Anokye Teaching Hospital, Ghana; a descriptive cross sectional study

**DOI:** 10.1186/s13104-017-3094-7

**Published:** 2017-12-19

**Authors:** Yaa Obirikorang, Christian Obirikorang, Enoch Odame Anto, Emmanuel Acheampong, Emmanuella Nsenbah Batu, Flora Macaulary, Christopher Kwaku Paavire, Bright Amankwaa

**Affiliations:** 1 Department of Nursing, Faculty of Health and Allied Sciences, Garden City University College (GCUC), Kenyasi, Kumasi, Ghana; 2 Department of Molecular Medicine, School of Medical Science, Kwame Nkrumah University of Science and Technology (KNUST), Kumasi, Ghana; 3 Department of Medical Laboratory Technology, Royal Ann College of Health, Atwima Manhyia, Kumasi, Ghana

**Keywords:** Bloodless medicine, Knowledge, Nurses

## Abstract

**Objective:**

This study assessed the knowledge of bloodless medicine (BM) among nurses at the medical and surgical directorate of Komfo Anokye Teaching Hospital. A paper questionnaire was administered to 322 nurses to obtain information on socio-demographic characteristics and knowledge of BM.

**Results:**

More than half (52.1%) of the nurses were aware of BM. The major source of information on BM was from the internet. Out of the study population, 90.1% knew iron therapy as BM strategy. The largest proportions of the participants (51.2%) had witnessed BM treatment at the medical and surgical directorate with the Tot’hema (44.6%) being the major used drug. Flu-like symptoms (37.0%) and organ damage (50.0%) were the commonly known side effects of BM respectively. Most of the participants (97.5%) knew that doctors request for blood transfusion. The major known reason for demand of BM was religious beliefs (53.7%). Higher percentages (71.9%) of the participants were not aware of bloodless surgery although the few who were aware, had heard of this through an internet search. Participants were generally aware of BM but less knowledgeable of specific components of BM. There is the need for prompt hospital and public health education through workshops and in-service training.

**Electronic supplementary material:**

The online version of this article (10.1186/s13104-017-3094-7) contains supplementary material, which is available to authorized users.

## Introduction

Bloodless medicine (BM) is one of the most important advances in the medical field both economically and technologically. Surgeons, physicians, and nurses began developing techniques for blood conservation in the early 1990’s as patients yearned to avoid blood transfusions due to the increasing risk of HIV or hepatitis contaminated blood, religious beliefs, or the harmful and weakening effects on the body’s defense system [1]. Blood transfusion is the transfer of blood and its products such as plasma, platelet clotting factors from one person to the other [2]. Some individuals view blood transfusion as something important when one is very ill. However, transfused blood can cause a number of haemolytic reactions due to incompatibility of blood [3].

Recent research has shown that allogeneic blood transfusion is similar to receiving an organ transplant, which is often associated with many risks and complications [2]. These practices have gained grounds in modern times all over the world including Africa and specifically Ghana, as blood continues to be scarce. Reports have shown that acute blood loss have been successfully treated with hyperbaric oxygen chambers [4]. Knowledge of blood conservation as part of blood management is essential to providing appropriate care and avoidance of unnecessary blood transfusion. There is paucity of published data on the knowledge of bloodless medicine among nurses. Therefore, this study explores the knowledge of BM among nurses at the Medicine and Surgery Departments of the Komfo Anokye Teaching Hospital (KATH), Ghana.

## Main text

### Study design and setting

This hospital-based cross-sectional descriptive study was conducted at KATH. It is located in Kumasi, the Regional capital of the Ashanti Region in Ghana with a total projected population of 4,780,380 (Ghana Statistical Service, 2010). It is the second largest hospital in Ghana and is the site for training doctors, biomedical scientists, nurses, health care assistants. Specialties in surgery, internal medicine, obstetrical and gynaecological, child health, oncology, family medicine and emergency medicine. The geographical location of the 1000 bed capacity of KATH, the road network of the country and commercial nature of Kumasi make the hospital accessible to all the areas that share the boundaries with Ashanti Region and others that are further away.

### Study population/selection of participants

Using a non-probability convenience sampling technique, a total of 322 nurses at the KATH were recruited from the period of January 2015 to February 2015. A quantitative research approach was used to assess the knowledge of BM. A structured questionnaire was developed based on the review of related journals. The pre-test or pilot study was conducted among 20 nurses to ascertain the contents and clarity of the questionnaire. The questions were piloted using a one-on-one interview with nurses by the study researchers who have been trained in data collection methods. Reliability coefficients ranging from 0.00 to 1.00, with higher coefficients indicating higher levels of reliability was used to determine the validity and the reliability of the questionnaire. The reliability coefficients for all the questions were 0.903. Changes were made to modify the questionnaire after the pilot study and the entire questionnaire was available in English. The questionnaire was then personally delivered to the nurses in paper form by the researchers. The questions were divided into two sections with close-ended questions. Section A: involved questions that elicited information on socio-demographic variables of the nurses such as age, years of experience, academic experience such as nurses with certificate, diploma degree, bachelor of science degree, and master of science degree: professional ranking such as principal nursing officer, senior nursing officer, nursing officer, senior staff nurse, staff nurse and enrolled nurse. Section B: included questions on the knowledge of BM comprising of yes/no questions and multiple choice formats. Nurses recruited for the study were instructed to choose multiple answers where applicable.

### Sample size

A total of 322 nurses from the medical and surgical directorate were recruited from a population of 2518 Nurses at Komfo Anokye Teaching Hospital using a assumed distribution response rate among the respondent of 60%, a precision of 5% at 95% confidence interval (z-score = 1.96).

### Inclusion/exclusion criteria

The study included all nurses present at the time of recruitment. The study excluded those who were unable to give informed consent. From the original 370 questionnaires distributed, a total of 322 were returned, deemed valid and analyzed.

### Statistical methods

The data entry and analysis were performed using IBM statistical package for social science (SPSS) version 20. Descriptive statistics were used and results obtained on the socio-demographic characteristic and knowledge of BM was presented into frequencies, proportions and percentages which were summarized into tables.

### Results

The study included 322 nurses for a response rate of 86.5%. Higher proportions of the participants were with age of 20–50 years. There were more females (94.2%) than male participants. Most (54.7%) of the participants were diploma holders followed by degree holders (30.4%), certificate (14.0%) and masters (0.9%). Majority of the nurses were senior staff (37.3%) followed by staff nurse (19.9%) and nursing officer (17.4%) (Table 1).

**Table 1:** Socio-demographic characteristics of nurses

Variables	Frequency (n = 322)	Percentages (%)
Age groups (years)		
20–30	210	65.2
31–40	75	23.1
41 and above	37	11.5
Gender		
Males	19	5.8
Females	303	94.2
Academic qualification		
Certificate	45	14.0
Diploma	176	54.5
Degree	98	30.5
Masters	26	0.8
Directorate		
Medicine	141	43.8
Surgery	181	56.2
Professional rankings		
Enrolled nurse	45	14.0
Staff nurse	64	19.9
Snr. staff nurse	120	37.3
Nursing officer	56	17.4
Snr. nursing officer	16	5.0
Principal nursing officer	21	6.5

Majority of the nurses (52.5%) have heard of BM and higher proportions (38.5%) of the nurses heard of BM through internet search while 23.1% heard of BM from their colleague nurses, 16.0% read from books or health publications, 13.0% heard from workshops and 9.5% heard of BM from the media (radio/TV). Out of the total participants, 68.2% knew the definition of BM. Two hundred and ninety nurses (90.1%) knew alternative medicine to blood transfusion. The commonest known alternative medicine reported by the participants were iron treatment (79.8%) followed by food supplements (63.3%), hormonal therapy (11.9%) and coagulation factors (4.6%) respectively (Table 2).

**Table 2:** Knowledge, awareness of bloodless medicine and sources of information

Variables	Frequency (n = 322)	Percentages (%)
Awareness of bloodless medicine		
Yes	169	52.5
No	153	47.5
Sources of information on bloodless medicine		
Radio/TV	16	9.5
Internet	65	38.5
Workshop	22	13.0
Books/health publications	27	16.0
A fellow nurse	39	23.1
Definition of bloodless medicine		
Treatment of patients with only plasma components of blood	13	4.1
Treatment of patients without packed red blood cell	32	9.9
Treatment of patient with only his/her own blood	11	3.3
Clinical care without allogeneic blood transfusion	146	45.5
Don’t know	120	37.2
Alternatives to blood transfusion		
Yes	290	90.08
No	32	9.92
Which alternative do you know?		
Iron treatment	257	79.8
Food supplements	204	63.3
Antifibrinolytics	–	–
Hormonal therapy	38	11.9
Coagulation factor	15	4.6

More than half the nurse (51.2%) had witness patients receiving BM. The most commonly used BM was Tot’hema (liquid iron supplement) (44.6%). Most of the participants (57.9%) did not know side effects/complications associated with BM. The most commonly known side effects/complication of BM were flu-like symptoms (98.6%), thrombosis (97.2%), skin reaction (83.3%) vomiting (87.5%) and hypertension (66.7%) (Table 3).

**Table 3:** Knowledge of the common type of BM, and side effects/complications of bloodless medicine used at KATH

Variable	Frequency (n = 322)	Percentage (%)
Have you witnessed a patient receiving bloodless medicine?		
Yes	165	51.2
No	157	48.8
Which of these medicines are commonly given?		
Interferon	42	12.9
Erythropoietin	99	30.6
Tothema	144	44.6
Novoseven	–	–
Tranexamic acid	62	19.3
Is bloodless medicine associated with complications?		
Yes	72	22.3
No	64	19.8
Don’t know	186	57.9
If yes which of these are known complication of bloodless medicine		
Hypertension	48	66.7
Skin reaction	60	83.3
Vomiting	63	87.5
Thrombosis	70	97.27
Flu-like symptoms	71	98.6

### Discussion

This study determined the knowledge of BM among nurses at KATH. Knowledge of BM among nurses has not been studied in the Ghanaian population. This study observed that majority of the nurses was aware of BM. It was interesting to know that majority of them had information on BM through the internet while others heard it from colleague nurses, health publications and the media (Table 2). This implies that there is lack of hospital-based education and in-service training on BM among study participants. Nursing interventions are based on conserving the patient’s energy and therefore creates healing environment [5]. However, this integrity cannot occur when there is lack of knowledge about therapeutic interventions.

The results of the study found majority of the nurses understood BM as “clinical care without allogeneic blood transfusion”. However, the proportions of the participants who did not know the definition of bloodless were comparable with those who knew the definition (37.2% vs. 45.5%). The probable explanation to the high knowledge of the definition of BM among nurse could be due to the internet search and the fact that as intellectuals, they are capable of understanding clinical terms. This study also observed that 90.1% of the participants knew alternatives to blood transfusion. The most commonly known alternative was iron treatment (79.8%). A previous study by Cuenca et al. [6] indicated that preoperative intravenous iron therapy reduced the need for allogeneic blood transfusion and hence could have accounted for it known use in the hospital of study. Another study by Muñoz et al. [7] showed that iron therapy is efficacious and safe compared allogeneic blood transfusion. However, it is noteworthy that many of the recommendations given for intravenous iron treatment could also be due to information from internet search.

Moreover, higher proportions of the participants had witnessed bloodless medicinal therapy. The most commonly known therapies by the participants were Tot’hema. The high usage of Tot’hema which largely contains iron, in this regard can be traced to the majority’s choice from the previous finding that iron treatment is the common alternative to bloodless medicinal therapy. A study by Feagan et al. [8] has also shown that erythropoietin was used as an alternative to prevent allogeneic blood transfusion in total hip joint arthroplasty. Moreover, allogeneic blood transfusions have been associated with increased morbidity and decreased survival; therefore knowledge of BM is needed in order to deliver holistic health care. Participants were less knowledgeable of the side effect associated with BM. For participants who knew bloodless medicine-associated side effect, majority of them knew flu-like symptom, followed by thrombosis, skin reaction, vomiting and hypertension. Previous study by Plješa [9] showed that hypertensive reaction, and thrombosis were possible complications of erythropoietin therapy, which is consistent with the findings of the present study. Flu-like symptoms, skin reaction and high blood pressure have also been associated with erythropoietin therapy in a study by Turner et al. [10].

The finding of the study revealed that more than half of the participants did not know risk factors of BM. Among those who knew the risk factors of BM, organ damage was the most common reported risk of BM followed by liver disease, severe exsanguination. The poor knowledge of the risk factors of BM may be explained by the fact that BM in contrast to blood transfusion is associated with little or no risk [11].

As expected, most of them agreed that doctors request blood transfusion. The results that doctors request for blood transfusion is due to the protocol that doctors are the only health professional who request for blood. Religious beliefs being the most commonly known factor is expected because the issue of bloodless medicine have been linked with religious beliefs by several studies [12–14]. Although BM and BS were created in response to the needs of Jehovah’s Witness patients, the interest in BM has expanded to people outside this group. In 2002, non-Witnesses comprised 25–30% of patients underwent BS [1]. The finding that, fear of complications was one of the factors associated with request for BM over blood transfusion is consistent with Shander [15]. In contrast to BM, majority of the participants were not aware of BS. The most common source of information was through internet search whiles others read it from medical journals, medical textbooks, media and seminars (Additional file 1: Table S1). Similar to the earlier finding on BM, most of the participants had information of BS through an internet search with a very few who had it from seminars. This presupposes that there is lack of in-service training, seminars and workshops on BM and surgery. However, nurses need to be knowledgeable on BM to remain current with prominent practice changes and regulatory practice guidelines.

### Conclusion

Nurses in the study were aware of BM; however they were less knowledgeable of the various side effects/complication and risk factors of BM. This study highlights the need for education of nurses on blood conservation methods and blood management for patients through incorporation of all facets of patient blood management in the curriculum of nursing schools. Moreover, prompt hospital and public health education on BM through seminars, workshops and in-service training is also required.

## Limitations

The major limitation of this study was the small sample size and the fact that this study used a cross-sectional design which does not make the findings of this study to be generalized. However, it would serve as a baseline for further research to use a large sample size coupled with inferential statistics to help make a general argument.

## Additional file

**Additional file 1: Table S1.** Knowledge of bloodless surgery risk factors of BM and reasons for demand of BM. Only 29.7% of the participants knew the risk factors of BM. The commonly known risk factors were organ injury/death (50.0%). 97.5% of the participants knew that more doctors other than nurse (3.3%) and patients (0.8%) request for blood transfusion. The most common reason for alternative medicine was religious beliefs (53.7%), followed by fear of transfusion complications (24.8%), ethical issues (9.9%), personal issue (9.0%) and economic issues (3.3%) respectively. Majority (71.9%) of the participants had never heard of BS. Most of the participants who were aware heard it from an internet search (97.8%), whiles others read it from the medical journal (14.4%), medical textbooks (52.2%), media (42.2%) and seminar (16.7%).

## Abbreviations

BM: bloodless medicine
BS: blood surgery
KATH: Komfo Anokye Teaching Hospital
