# Supplementary material for: Knowledge of bloodless medicine among nurses at the Medical/Surgical Directorate of Komfo Anokye Teaching Hospital, Ghana; a descriptive cross sectional study
Source: BMC Res Notes. 2017 Dec 19;10:747. doi: 10.1186/s13104-017-3094-7 (PMC5735550; doi:10.1186/s13104-017-3094-7)
Supplement: Supplementary file 1 — Additional file 1: Table S1. Knowledge of bloodless surgery risk factors of BM and reasons for demand of BM. Only 29.7% of the participants knew the risk factors of BM. The commonly known risk factors were organ injury/death (50.0%). 97.5% of the participants knew that more doctors other than nurse (3.3%) and patients (0.8%) request for blood transfusion. The most common reason for alternative medicine was religious beliefs (53.7%), followed by fear of transfusion complications (24.8%), ethical issues (9.9%), personal issue (9.0%) and economic issues (3.3%) respectively. Majority (71.9%) of the participants had never heard of BS. Most of the participants who were aware heard it from an internet search (97.8%), whiles others read it from the medical journal (14.4%), medical textbooks (52.2%), media (42.2%) and seminar (16.7%). [file 13104_2017_3094_MOESM1_ESM.docx]

**Table S1: Knowledge of bloodless surgery risk factors of BM and reasons for demand of BM**

| Variables | Frequency (n=322) | Percentages (%) |
| --- | --- | --- |
| Knowledge on risk factors bloodless medicine |  |  |
| Yes | 96 | 29.70% |
| No | 45 | 14.0% |
| Don't Know | 181 | 56.2% |
| If yes which of these are known risk factors of BM |  |  |
| Organ injury/death | 161 | 50.0% |
| Liver disease | 90 | 27.8% |
| Severe Exsanguinations | 45 | 13.9% |
| Severe blood clot | 86 | 25.0% |
| Heart/blood vessel diseases | 72 | 22.2% |
| Personality who request for blood transfusion |  |  |
| Patients | 3 | 0.80% |
| Relatives | - | - |
| Religious | - | - |
| Nurse | 11 | 3.30% |
| Doctor | 314 | 97.50% |
| Reason accounting for demand of BM |  |  |
| Personal | 29 | 9.0% |
| Religious belief | 173 | 53.7% |
| Medical/Ethical | 32 | 9.9% |
| Economic | 11 | 3.3% |
| Fear of blood transfusion complications | 80 | 24.8% |
| Have you heard of bloodless surgery |  |  |
| Yes | 90 | 28.1% |
| No | 232 | 71.9% |
| If yes where was your source of information |  |  |
| Medical journal/Brochure | 13 | 14.4% |
| Medical Textbook | 47 | 52.2% |
| Workshop/Seminar | 19 | 16.7% |
| Radio/TV | 38 | 42.2% |
| Internet | 88 | 97.8% |
